# Supplementary material for: Base-editing corrects metabolic abnormalities in a humanized mouse model for glycogen storage disease type-Ia
Source: Nat Commun. 2024 Nov 10;15:9729. doi: 10.1038/s41467-024-54108-1 (PMC11551175; doi:10.1038/s41467-024-54108-1)
Supplement: Supplementary file 1 — Supplementary Information [file 41467_2024_54108_MOESM1_ESM.pdf]

Supplementary information for

Base-editing corrects metabolic abnormalities in a humanized mouse model for glycogen storage disease type-Ia

Irina Arnaoutova[1], Yvonne Aratyn-Schaus[2], Lisa Zhang[1], Michael S. Packer[2], Hung-Dar Chen[1], Cheol Lee[1], Sudeep Gautam[1], Francine M. Gregoire[2], Dominique Leboeuf[2], Steven Boule[2], Thomas P. Fernandez[2], Victoria Huang[2], Lo-I Cheng[2], Genesis Lung[2], Brianna Bannister[2], Jeremy Decker[2], Thomas Leete[2], Lan S. Shuang[2], Caroline Bock[2], Prachi Kothiyal[2], Phil Grayson[2], Ka W. Mok[2], Jeffrey J. Quinn[2], Lauren Young[2], Luis Barrera[2], Giuseppe Ciaramella[2], Brian C. Mansfield[1], and Janice Y. Chou[1*]

Correspondence to: Janice Y. Chou; chouja@mail.nih.gov

Supplementary Figures and Legends

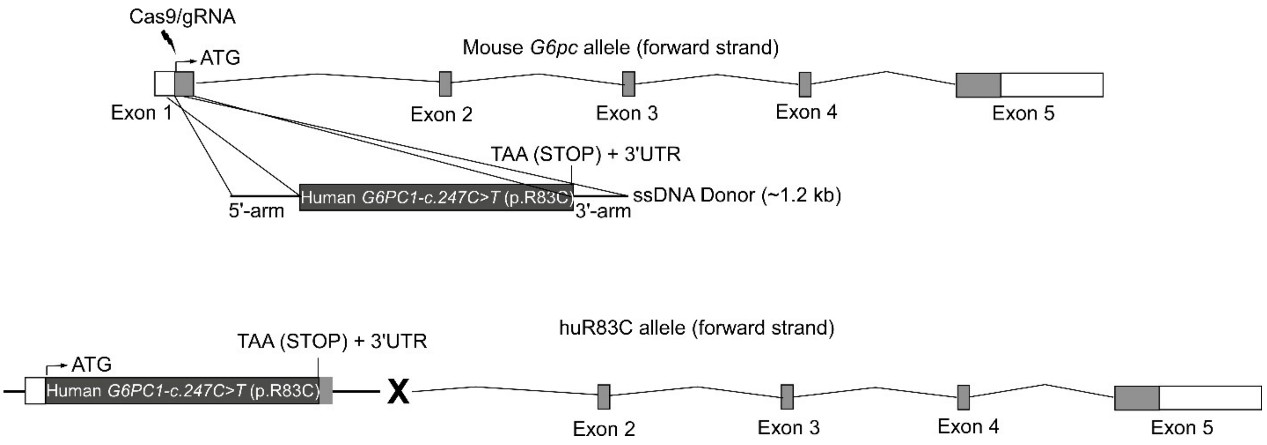

Supplementary Fig. 1. The human *G6PC1-c.247C>T* cDNA knock-in strategy. The generation of the heterozygous (mR83/huR83C) mice carrying one functional mouse *G6pc*-R83 allele and one human *G6PC1*-R83C (*G6PC1*-c.247C>T) variant allele involved the simultaneous knock-out of a mouse *G6pc* allele by the knock-in of a human *G6PC1*-c.247C>T cDNA. The on-target insertion of the human *G6PC1*-c.247C>T cDNA encoding the p.R83C variant was designed to disrupt the mouse *G6pc* gene by co-opting the *G6pc* translation initiation site for expression of the human *G6PC1*-c.247C>T cDNA, which included a 3’ UTR and poly(A) signal sequence, upstream of the remainder of the *G6pc* gene.

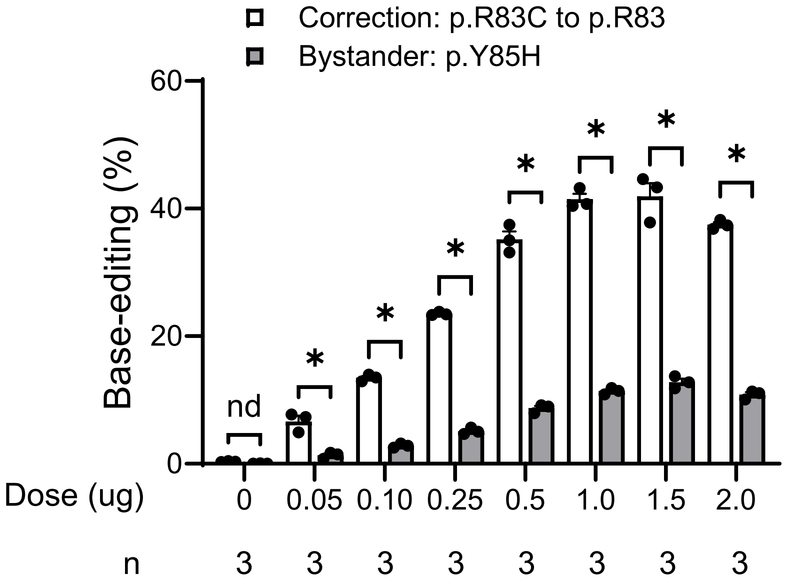

Supplementary Fig. 2. In vitro correction of the *G6PC1*-R83C allele by base editing. Primary human hepatocyte co-cultures, harboring the *G6PC1*-c.247C>T mutation within the *G6PC1* transgene via lentiviral transduction, were dosed with BEAM-301 mRNA and gRNA constituents via lipofection transfection. Cell lysates were generated one week post transfection, and the target site sequence was analyzed via NGS to determine the rate for correction of the p.R83C (*G6PC1- c.247C>T*) variant to wild-type p.R83 (*G6PC1- c.247C*) and introduction of the p.Y85H bystander variant. The study was run in triplicate, with n = 3 replicates, and the amount of total RNA (mRNA and gRNA) that was dosed is noted across a dose range. Statistics were performed using a two-tailed unpaired T test. Data are presented as Mean values ± SEM, and individual data points for each replicate are displayed. * denotes $p < 0.01$.

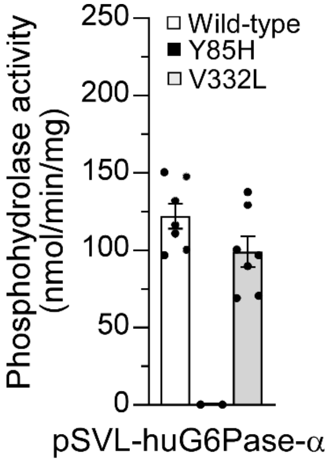

Supplementary Fig. 3. Characterization of human G6Pase-α variants by i*n vitro* expression. Phosphohydrolase activity of human (hu) G6Pase-α wild-type (n = 7), Y85H (n = 2), and V332L (n = 7) following transfection of the respective pSVL construct into COS-1 cells. The pSVL-huG6Pase-α-wild-type vector containing the entire coding region of *G6PC1* cDNA at nucleotides 1 to 1074 was used as a template to generate the pSVL-huG6Pase-α-Y85H and the pSVL-huG6Pase-α-V332L constructs by PCR-directed mutagenesis as previously described[30]. The pSVL-huG6Pase-α-Y85H construct displayed non-detectable phosphohydrolase activity. The pSVL-huG6Pase-α-V332L construct retained, on average, 81% of phosphohydrolase activity displayed by the pSVL-huG6Pase-α-wild-type construct, but the differences in phosphohydrolase activity between huG6Pase-α-wild-type and huG6Pase-α-V332L were not statistically significant. Statistics were performed using a two-tailed unpaired T test. Data are presented as Mean values ± SEM.

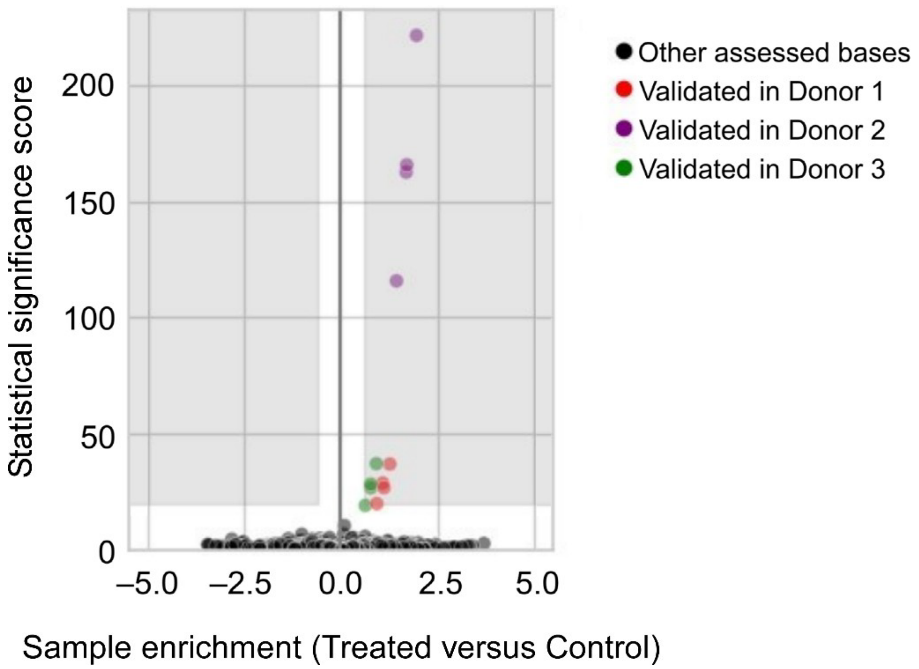

Supplementary Fig. 4. Off-target editing analysis. The primary human hepatocytes obtained from three donors (shown in three colors) were used for off-target editing analysis (Supplementary Table 2). Each individual point represents a single base in a candidate off-target site compared between treated and untreated samples in a single donor. The shaded area corresponds to the designated zone of statistical significance (-log10 (*P*) ≥20, sample enrichment ≥1.5). Treated vs. control sample enrichment was calculated as log2 (% in treated sample/% in control sample). Positive values indicate enrichment in the treated sample relative to the control sample, whereas negative values indicate enrichment in the control sample over the treated sample. The statistical significance score corresponds to -log10 (*P*)*, where P is calculated by* Fisher's exact test. All statistics were generated using python3.8.12, numpy1.21.2. Significant A:T to G:C sites were manually reviewed both to validate true-positive off-target sites and ensure no false-positives. One true-positive off-target site was observed (the 12 red, green, and purple datapoints are consistent with one site; 3 donors with 2 treated replicates per donor were compared for enrichment against each of the 2 donor-matched, untreated background control samples).

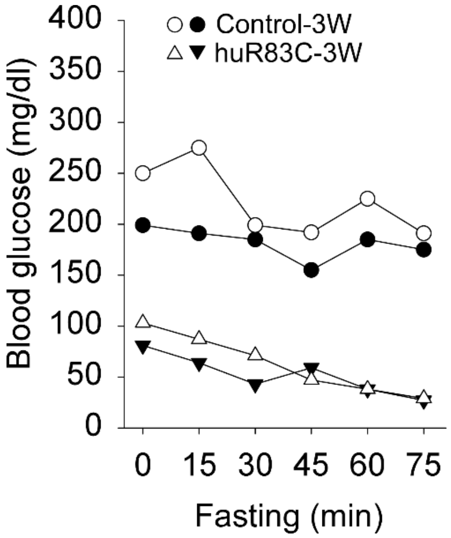

Supplementary Fig. 5. Fasting blood glucose profile of the 3-week-old untreated huR83C mice. Representative fasting glucose profiles of two 3-week-old control mice and two untreated huR83C mice.
