# Supplementary material for: Base-editing corrects metabolic abnormalities in a humanized mouse model for glycogen storage disease type-Ia
Source: Nat Commun. 2024 Nov 10;15:9729. doi: 10.1038/s41467-024-54108-1 (PMC11551175; doi:10.1038/s41467-024-54108-1)
Supplement: Supplementary file 2 — Description of Additional Supplementary Files [file 41467_2024_54108_MOESM2_ESM.pdf]

Description of Additional Supplementary Files

Supplementary Data 1- Guide RNA and mRNA sequences

Supplementary Data 2- Off-target editing analysis table
