# Supplementary material for: Base-editing corrects metabolic abnormalities in a humanized mouse model for glycogen storage disease type-Ia
Source: Nat Commun. 2024 Nov 10;15:9729. doi: 10.1038/s41467-024-54108-1 (PMC11551175; doi:10.1038/s41467-024-54108-1)
Supplement: Supplementary file 5 — Reporting summary [file 41467_2024_54108_MOESM5_ESM.pdf]

nature portfolio

Corresponding author(s): Janice Y. Chou NCOMMS-24-19638A

Last updated by author(s): Sep 7, 2024

Reporting Summary

Nature Portfolio wishes to improve the reproducibility of the work that we publish. This form provides structure for consistency and transparency in reporting. For further information on Nature Portfolio policies, see our Editorial Policies and the Editorial Policy Checklist.

Statistics

For all statistical analyses, confirm that the following items are present in the figure legend, table legend, main text, or Methods section.

n/a	Confirmed	
[ ]	[x]	The exact sample size (*n*) for each experimental group/condition, given as a discrete number and unit of measurement
[x]	[ ]	A statement on whether measurements were taken from distinct samples or whether the same sample was measured repeatedly
[ ]	[x]	The statistical test(s) used AND whether they are one- or two-sided<br>*Only common tests should be described solely by name; describe more complex techniques in the Methods section.*
[x]	[ ]	A description of all covariates tested
[x]	[ ]	A description of any assumptions or corrections, such as tests of normality and adjustment for multiple comparisons
[ ]	[x]	A full description of the statistical parameters including central tendency (e.g. means) or other basic estimates (e.g. regression coefficient) AND variation (e.g. standard deviation) or associated estimates of uncertainty (e.g. confidence intervals)
[x]	[ ]	For null hypothesis testing, the test statistic (e.g. *F*, *t*, *r*) with confidence intervals, effect sizes, degrees of freedom and *P* value noted<br>*Give P values as exact values whenever suitable.*
[x]	[ ]	For Bayesian analysis, information on the choice of priors and Markov chain Monte Carlo settings
[x]	[ ]	For hierarchical and complex designs, identification of the appropriate level for tests and full reporting of outcomes
[x]	[ ]	Estimates of effect sizes (e.g. Cohen's *d*, Pearson's *r*), indicating how they were calculated

Our web collection on statistics for biologists contains articles on many of the points above.

Software and code

Policy information about availability of computer code

Data collection	*Provide a description of all commercial, open source and custom code used to collect the data in this study, specifying the version used OR state that no software was used.*
Data analysis	The statistical analyses reported are two-tailed unpaired T tests performed using the GraphPad Prism Program, version 10.2.2 (GraphPad Software).

For manuscripts utilizing custom algorithms or software that are central to the research but not yet described in published literature, software must be made available to editors and reviewers. We strongly encourage code deposition in a community repository (e.g. GitHub). See the Nature Portfolio guidelines for submitting code & software for further information.

Data

Policy information about availability of data

All manuscripts must include a data availability statement. This statement should provide the following information, where applicable:

- Accession codes, unique identifiers, or web links for publicly available datasets
- A description of any restrictions on data availability
- For clinical datasets or third party data, please ensure that the statement adheres to our policy

All data supporting the findings described in this manuscript including the mRNA encoding the ABE are available in the Article, Supplementary Information, and Source data file published alongside the paper. The NGS sequencing data is currently being deposited in a publicly accessible database. We will send Nature

Communication the accession code when we receive it.

Research involving human participants, their data, or biological material

Policy information about studies with human participants or human data. See also policy information about sex, gender (identity/presentation), and sexual orientation and race, ethnicity and racism.

Reporting on sex and gender	For the human primary hepatocytes and the B-lymphocyte cell line (GM11468, heterozygous for the G6PC1-c.247C>T/p.R83C allele) used in this study, the sex of the donor was not considered in the study design. GSD-Ia is an autosomal recessive disorder and GSD-Ia patients do not display phenotypic variation between gender/sex.
Reporting on race, ethnicity, or other socially relevant groupings	Not applicable.
Population characteristics	The human B-lymphocyte cell line (GM11468, Coriell) used in the Digenome-seq assay is derived from a compound heterozygous female GSD-Ia patient carrying the p.R83C allele, the subject of this study, along with the p.Q347X allele.<br><br>Given that the liver is the target organ of base editing and that GSD-Ia patient-derived hepatocytes harboring the G6PC1-c.247C>T/p.R83C variant are not available, primary human hepatocytes obtained from commercially available donors who did not have disclosed liver disease were selected for rhAmp-seq analysis.
Recruitment	The criteria for selection of the primary human hepatocytes were based on commercial availability; appropriate characterization, supported by expression of hepatic markers; and cell viability. To avoid bias, three unrelated donors were incorporated into the study design, and the off-target profiles confirmed across all donors.
Ethics oversight	The primary human hepatocytes were provided by BioVT. BioIVT collects high quality, fully characterized, diseased and normal tissue from live donors, sourced from a large network of collection sites and research hospitals, both domestic and foreign. BioIVT ensures that the tissue collection network operates in a fully ethical and transparent manner. IRB approved protocols and collection sites - De-identified specimens - International research network operates to domestic standards and BioIVT quality policies.

Note that full information on the approval of the study protocol must also be provided in the manuscript.

Field-specific reporting

Please select the one below that is the best fit for your research. If you are not sure, read the appropriate sections before making your selection.

☒ Life sciences ☐ Behavioural & social sciences ☐ Ecological, evolutionary & environmental sciences

For a reference copy of the document with all sections, see nature.com/documents/nr-reporting-summary-flat.pdf

Life sciences study design

All studies must disclose on these points even when the disclosure is negative.

Sample size	No statistical sample size calculation was performed to predetermine sample size. The huR83C mice manifest a GSD-Ia phenotype with hallmark of fasting hypoglycemia. The 3-week survival rate of the huR83C mice is low (39%) , and the 8-week survival rate is 0%. In this study, we expected BEAM-301, the therapeutic base editing reagent to improve the 3-week survival rate of the affected mice to 100%. For our short-term proof of concept studies, we used a minimum of 8 affected (huR83C) and 8 control mice with the expectation this would produce statistically significant values if successful. Based on the outcomes of those studies, the long-term therapeutic studies (53 weeks) used 9 to 19 huR83C and 14 to 16 control mice to strengthen the statistical significance. Specific sample sizes are provided for each experiment.
Data exclusions	In our studies of newborn (NB) affected huR83C mice treated by systemic administration of the BEAM-301, at a dose level of 1.5 mg/kg (301H), the metabolic correction was assessed at 3 weeks of age. Of the eight treated mice, two expressed background hepatic and renal G6Pase-α activity, and genomic analysis confirmed the absence of base-editing in their livers. Therefore, they were excluded from further analysis, and this was clearly stated in Results under Pathophysiology of 301H-dosed huR83C mice at 3 weeks of age.
Replication	Our genetic editing studies were conducted using two different dosages of the BEAM-301 base editing reagent via a single systemic administration either in newborn (NB) mice or at 3 weeks (3W) of age. The outcomes were monitored at three different end points, 3 weeks, 8 weeks, and 53 weeks and all data and replications are reported and consistent.
Randomization	All mice were chosen randomly from their appropriate litters (homozygous affected mice, wild-type mice, and unaffected heterozygous mice). Allocation of mice into the experimental groups was completely random.
Blinding	The investigators were blinded to group allocation during data collection and analysis.

Reporting for specific materials, systems and methods

We require information from authors about some types of materials, experimental systems and methods used in many studies. Here, indicate whether each material, system or method listed is relevant to your study. If you are not sure if a list item applies to your research, read the appropriate section before selecting a response.

Materials & experimental systems

n/a	Involved in the study
☒	☐ Antibodies
☐	☒ Eukaryotic cell lines
☒	☐ Palaeontology and archaeology
☐	☒ Animals and other organisms
☒	☐ Clinical data
☒	☐ Dual use research of concern
☒	☐ Plants

Methods

n/a	Involved in the study
☒	☐ ChIP-seq
☒	☐ Flow cytometry
☒	☐ MRI-based neuroimaging

Eukaryotic cell lines

Policy information about cell lines and Sex and Gender in Research

Cell line source(s)	The human B-lymphocyte cell line GM11468 is a human B-lymphocyte cell line heterozygous for the G6PC1 c.247C>T allele obtained from Coriell Institute (Camden, NJ). The DNA isolated from GM11468 was used for Digenome-seq which has been clearly described in Methods.
Authentication	Cell line authentication was provided by the supplier and not independently confirmed in this study
Mycoplasma contamination	Cell lines were provided by the supplier as mycoplasma free and this was not independently confirmed
Commonly misidentified lines (See ICLAC register)	Not applicable.

Animals and other research organisms

Policy information about studies involving animals; ARRIVE guidelines recommended for reporting animal research, and Sex and Gender in Research

Laboratory animals	The laboratory animals used are mice in a mixed background of C57BL/6 (50%) and 129S4/SvJaeJ (50%). The ages of the mice studied were from newborn to 53 weeks of age.
Wild animals	The study did not involve wild animals.
Reporting on sex	Sex was not considered in the study design. GSD-Ia is an autosomal recessive disorder and neither GSD-Ia patients nor mice display phenotypic variation between gender/sex. Therefore, sex was not considered in this preclinical study design.
Field-collected samples	The study did not involve samples collected from the field
Ethics oversight	Animal studies were conducted either under an animal protocol approved by the Animal Care and Use Committee at Eunice Kennedy Shriver National Institute of Child Health and Human Development or an animal protocol approved by the Institutional Animal Care and Use Committee at University of Massachusetts Medical School and CRADL® (Charles River Accelerator and Development Lab) in Cambridge, Massachusetts. All animal procedures have been done according to the institutional guidelines and approved by the local ethics committee.

Note that full information on the approval of the study protocol must also be provided in the manuscript.

Plants

Seed stocks	*Report on the source of all seed stocks or other plant material used. If applicable, state the seed stock centre and catalogue number. If plant specimens were collected from the field, describe the collection location, date and sampling procedures.*
Novel plant genotypes	*Describe the methods by which all novel plant genotypes were produced. This includes those generated by transgenic approaches, gene editing, chemical/radiation-based mutagenesis and hybridization. For transgenic lines, describe the transformation method, the number of independent lines analyzed and the generation upon which experiments were performed. For gene-edited lines, describe the editor used, the endogenous sequence targeted for editing, the targeting guide RNA sequence (if applicable) and how the editor was applied.*
Authentication	*Describe any authentication procedures for each seed stock used or novel genotype generated. Describe any experiments used to assess the effect of a mutation and, where applicable, how potential secondary effects (e.g. second site T-DNA insertions, mosiacism, off-target gene editing) were examined.*
